# Comparative study of chemical pathology sample collection tubes at the largest hospital in South Africa

**DOI:** 10.5937/jomb0-27216

**Published:** 2021-09-03

**Authors:** Siyabonga P. Khoza, Sarah Ford, Ernest P. Buthelezi, Donald M. Tanyanyiwa

**Affiliations:** 1 University of the Witwatersrand, Johannesburg, South Africa; 2 National Health Laboratory Service, Department of Chemical Pathology, Johannesburg, South Africa; 3 Gauteng Department of Health, Johannesburg, South Africa; 4 Sefako Makgatho University of Health Sciences, Pretoria, South Africa

**Keywords:** BarricorTM, blood collection devices, serum separator tubes, BarricorTM, uređaji za vađenje krvi, epruvete za odvajanje seruma

## Abstract

**Background:**

Barricor^TM^ Lithium heparin plasma tubes are new blood tubes that have been introduced to overcome the effects of gel in serum separator tubes (SST) and the shortcomings of standard Lithium heparin plasma. We aimed to evaluate Barricor^TM^ tubes as an alternative to serum separator tubes and compare the stability between the tubes.

**Methods:**

Forty-four paired samples were collected using both Barricor^TM^ and SST. We compared five analytes at baseline (<6 h) and after every 24 h using the PassingBablok and Bland-Altman plots. Aspartate aminotransferase (AST), potassium (K), phosphate (PO4) , lactate dehydrogenase (LDH), and creatinine were analysed in both tubes. We calculated the percentage difference for each analyte between the baseline and time intervals to assess analyte stability. The percentage difference was compared to the desirable specification for bias and reference change value (RCV).

**Results:**

All analytes were comparable at baseline. Statistical differences (p<0.001) became evident after 24 h. PO4, K, and creatinine had a mean difference that exceeded the desirable specification for bias (-9.59%, - 9.35%, and -4.59%, respectively). Potassium was stable up to 24 h in both tubes. LDH showed better stability in SST (144 h vs 96 h). PO4 concentrations were more stable in both tubes with the SST (96 h vs 72 h). Creatinine and AST had the longest stability in both tubes compared to other analytes (144 h).

**Conclusions:**

Data demonstrated variability and similarities in analyte concentrations and stability, respectively, in both tubes.

## Abstract (sr)

**Uvod:**

BarricorTM litijumske heparinske epruvete za prikupljanje plazme su nove epruvete za krv koje su uvedene kako bi se prevazišli problemi uticaja gela u epruvetama za odvajanje seruma (SST) i nedostaci standardne litijumheparinske epruvete. Cilj NAM je bio da ocenimo BarricorTM epruvete kao alternativu serumskim separatorima i uporedimo stabilnost između epruveta.

**Metode:**

Prikupljena su četrdeset i četiri uparena uzorka pomoću epruveta BarricorTM i SST. Upoređivali smo pet analita na početku (<6 h) i nakon svaka 24 sata, koristeći Passing-Bablok i Bland-Altman metode. U obe epruvete su analizirani aspartat aminotransferaza (AST), kalijum (K), fosfat (PO4), laktat dehidrogenaza (LDH) i kreatinin. Izračunali smo procentualnu razliku za svaki analit između početnog vremena i vremenskih intervala da bismo procenili stabilnost analita. Procentualna razlika je upoređena sa poželjnom specifikacijom sklonosti i referentnih vrednosti (RCV).

**Rezultati:**

Svi analiti su u početku bili uporedivi. Statističke razlike (p<0,001) su postale očigledne nakon 24 sata. PO4, K i kreatinin su imali srednju razliku koja je premašila poželjnu specifikaciju sklonosti (-9,59%, -9,35% i -4,59%, redom). Kalijum je bio stabilan do 24 sata u obe epruvete. LDH je pokazao bolju stabilnost u epruveti SST (144 h prema 96 h). Koncentracije PO4 su bile stabilnije u obe epruvete sa SST (96 h prema 72 h). Kreatinin i AST su imali najdužu stabilnost u obe epruvete u poređenju sa drugim analitima (144 h).

**Zaključak:**

Podaci su pokazali promenljivost i sličnost u koncentraciji i stabilnosti analita, redom, u obe epruvete.

## Introduction

Rapid analysis of samples is an essential step necessary to ensure the quality and integrity of laboratory results. Improvement of the analytical phase of the laboratory testing process has resulted in a few errors in this phase. Approximately 60% of errors occur in the pre-analytical phase; therefore, it is critical to minimise their effects on the quality of results [1]. The influence of blood collection devices on laboratory results is often overlooked [1].

It is known that blood cells (red, white, and platelets) undergo lysis during storage, leading to an increased release of lactate dehydrogenase (LDH), potassium, and phosphate, while metabolically active cells continue to consume glucose [2]. Babic et al. [3] demonstrated that the forces exerted on red blood cells caused them to cross the gel barrier post centrifugation, thus increasing intracellular analytes such as potassium. Furthermore, separator gel tubes are also found to release small gel materials into the serum or plasma. These gel particles have been associated with interference in immunoassays, sample probes, and electrode surfaces [4].

Although serum is the most commonly used specimen type for analysis of biochemical tests, plasma has some benefits such as no need for lengthy delays required for clotting, shorter centrifugation time, and therefore reduced turnaround time [5]. Due to the lack of a complete barrier between the cells and the supernatant, some have suggested that transferring plasma to a secondary tube may overcome this problem; however, this is impractical for a busy laboratory [5].

BD Barricor^TM^ lithium heparin (LH) plasma Vacutainer^®^ collection tubes have been introduced to overcome the effects caused by the gel in SST tubes and the shortcomings of standard lithium heparin plasma. Barricor^TM^ LH plasma tubes rely on the mechanical separation of plasma from cells. The tube has a mechanical stopper which has a large elastomer top, which allows stretching and manipulation during centrifugation. During centrifugation, the mechanical separator/elastomer stretches and creates small channels around it, allowing blood cells to sediment out of the plasma to the bottom. Once the centrifugation speed slows down, the elastomer returns to its original position, creating a stable and rigid seal in the process (www.barricor.bd.com/us/how-bd-barricor-tubeworks.xml). The mechanical stopper offers chemical inert separation, therefore not reacting with the analytes, and thus longer sample stability (www.barricor.bd.com/us/how-bd-barricor-tubeworks.xml) [6]. It offers a shorter centrifugation time, which is ideal for urgent samples and may reduce turnaround time (3 min vs 10 min) [6].

Füzéry et al. [7] demonstrated that the Barricor tube is a good alternative to traditional plasma separator tubes (PST), although the study only looked at the AccuTnI +3 assay. Another study demonstrated improved lithium heparin from the Barricor^TM^ tube when compared to PST and SST tubes. This was evident by the reduced red cell numbers and mild changes in potassium, LDH, and phosphate resulting from cellular lysis and utilisation of glucose during storage [3] [8] [9]. Given the lack of sufficient studies, we aimed to evaluate the BD Barricor^TM^ tube as an alternative to serum separator tubes. Furthermore, the aim was to compare the stability of BD Barricor^TM^ tubes to SST over a period of time for routine testing.

## Material and Methods

### Subjects and collection tubes

The study was conducted at the Lenasia District Hospital between May and June 2019. This is a district hospital that sends blood samples to the Chris Hani Baragwanath Academic Hospital Laboratory. The study obtained institutional ethics approval (clearance certificate no. M181134). Informed consent was obtained from all participants (≥18 years). Nurses collected blood samples from the same subject into two tubes, SST tube first followed by the Barricor^TM^ tube. This gave rise to paired samples from 49 participants. Five participants were excluded because samples were haemolysed (3 participants), and two were missing Barricor^TM^ tubes.

Both Barricor^TM^ tubes (Lot: 8183819, Becton Driven (BD), Franklin Lakes, USA) and SST tubes (Lot: 8330585, BD) were mixed as per recommendation by the manufacturer and centrifuged within 15 min, while SST tubes were allowed to clot for 30 min prior to centrifugation at 3000x g for 5 min using a Drucker Apex Dash 24 swing-bucket rotor at room temperature. Specimens were kept upright at room temperature until analysis.

### Instrument and sample analysis

After centrifugation, samples were measured for aspartate aminotransferase (AST), potassium (K), lactate dehydrogenase (LDH), phosphate (PO_4_), and creatinine on the Roche Cobas 8000 system. Potassium was measured using an indirect ion-selective electrode method, phosphate was measured using molybdate ultraviolet, while LDH, AST, and creatinine were analysed using enzymatic methods. The samples were analysed at 0 times (baseline which from time of collection was 6 h post collection), 24, 48, 72, 96, 120, and 144 h post collection. Samples were stored at 4°C between time intervals from the primary tubes. Serum indices were performed spectrophotometrically, and those that were haemolysed were excluded from further analysis. Internal and external quality controls were acceptable during the study period, and there were no changes noted.

### Analyte stability assessment

For stability assessment, a comparison of the analytes values from both tubes was assessed at specified time intervals. This was compared to the initial value obtained to assess stability over time. The analytes that were assessed include creatinine, potassium (K), inorganic phosphorus (PO 4), lactate dehydrogenase (LDH), and aspartate aminotransferase (AST). Plasma and serum were left in the primary tube and kept at 4°C for seven days. Before each measurement, samples were taken out of the fridge and kept at room temperature for at least 30 min before analysis.

### Statistical analyses

Data are reported as the median with interquartile range (IQR). A comparison between the tubes was analysed using Passing-Bablok and difference plots. The intercepts and slopes were considered nonsignificant when the 0 or 1 values fell within a 95% confidence interval (CI), respectively. The Spearman correlation coefficient was performed to assess the correlation between the tubes. The difference between the tubes at a one-time point collection was calculated as difference (%) = (concentration in Barricor^TM^ concentration in SST tube)/concentration in SST tube*100 using Microsoft Excel 2010. The difference was compared to the desirable total allowable error (TE_a_) [10]. Furthermore, the scatter of differences was visualised using Bland-Altman plots, and the mean difference% reported with 95% CI (mean difference±1.96 SD). The limit of agreements (LOA) was calculated as the interval defined by±1.96 standard deviations (SD) between the tubes at which 95% of the differences lie. Also, the mean differences were compared with desirable bias using biological variation data from the European Federation of Clinical Chemistry and Laboratory Medicine (EFLM) [10] for clinical significance analysis. Desirable specifications for imprecision and bias were calculated as follows: Imprecission_desirable_= 0.5×CV_within-subject_ and Bias_desirable_= 0.25×(CV_within-subject_^2^ +CV_between-subject_^2^)^½^. The total error (TE) was calculated as follows:

TE_a_= 1.65(0.5×CVI) + 0.25(CVI^2^ +CVG^2^)^½^.

The difference in concentration over time in one tube was calculated as the difference (%)= (concentration at time point concentration at baseline)/concentration at baseline*100, and the difference was compared to the desirable specification for the coefficient of variation for bias (CV_b_) [10]. The analyte was considered unstable if the difference exceeded CV b for five parameters at a particular time point. Besides, the reference change values (RCV) for each analyte were estimated using the following formula: RCV (%)=2^1/2^*Z*(CV_a_^2^+CV_I_^2^)^1/2^ [11]. Z is 1.96 for the two-sided approach for 95%, coefficient of variation (CV_a_), laboratory analytical imprecision for a 6-month period, and CV_I_ within-in subject variation was obtained from EFLM biological variation [10]. If the percentage difference between the sequential results exceeds the RCV, the difference is considered clinically significant. Passing Bablok, Bland-Altman plots, and Spearman correlation were performed using Analyze-it (Microsoft Excel 2010 vers 5.11)

## Results

Baseline measurements of the concentrations of the different analytes in the SST and Barricor^TM^ tube are compared in Table 1. Passing-Bablok regression analysis showed no significant bias for most analytes. Potassium and PO_4_ exceeded the total allowable error. A strong correlation was found between the tubes investigated, and Spearman R_s_ correlation ranged from 0.730 to 0.984, while potassium showed the least correlation. Creatinine was found to have the strongest correlation with R_s_ of 0.984. Statistically, differences in median concentration for the five analytes were detected (P-value <0.001). Also, the mean difference between the tubes for most analytes at baseline did not exceed the total allowable error (TE_a_), except for the clinically significant difference for potassium.

**Table 1:** Comparison between serum separator tubes and Barricor^TM^ lithium heparin tubes for the five chemistry analytes SST = serum separator tube; AST = Aspartate aminotransferase; K = Potassium; LDH = Lactate dehydrogenase; CI = Confidence intervals; ^a^The baseline value considered serum/plasma measured less 6h post collection; ^b^Passing-Bablok regression analysis; ^c^Spearman correlation coef- ficient; ^d^Wilcoxon matched paired test, statistical significant if P< 0.05; ^e^Differences are calculated as = (Concentration in BD Barricor – Concentration in SST)/Concentration in SST*100; ^f^TEa = 1.65(0.5xCVI) + 0.25(CVI^2^+CVG^2^)^½^ using biological variation data from EFLM

			SST tube	Barricor						
Analyte	Unit	Sample Number	Median Baseline valu^a^ (IQR)	Median Baseline valu^a^ (IQR)	PB^b^ 95% CI Intercept	PB^b^ 95% CI Slope	Rs^c^ 95% CI	P^d^	Mean Difference (%)^e^	TEa^f^
AST	U/L	44	22 (17–30)	23 (18–30)	1.20 (-0.195–2.516)	0.962 (0.9032–1.0209)	0.919 (0.855–1.021)	<0.001	1.20	13.8
K	mmol/L	44	4.3 (4.0–4.5)	3.9 (3.6–4.2)	-0.669 (-1.588–0.700)	1.067 (0.923–1.289)	0.730 (0.553–0.844)	<0.001	-8.68	4.85
Phosphate	mmol/L	44	1.06 (0.93–1.18)	0.95 (0.84–1.05)	-0.100 (-0.1802–0.016)	1.00 (0.889–1.080)	0.917 (0.853– 0.954)	<0.001	-8.79	8.37
LDH	U/L	44	198 (178–240)	210 (177–237)	3.00 (-20.417–20.431)	0.979 (0.896–1.081)	0.862 (0.760–0.923)	<0.001	-0.70	7.67
Creatinine	mmol/L	44	77 (63–89)	74 (58–87)	-3.00 (-5.429–0.816)	1.00 (0.947–1.029)	0.984 (0.971–0.991)	<0.001	-4.2	7.37

**Figure 1:**
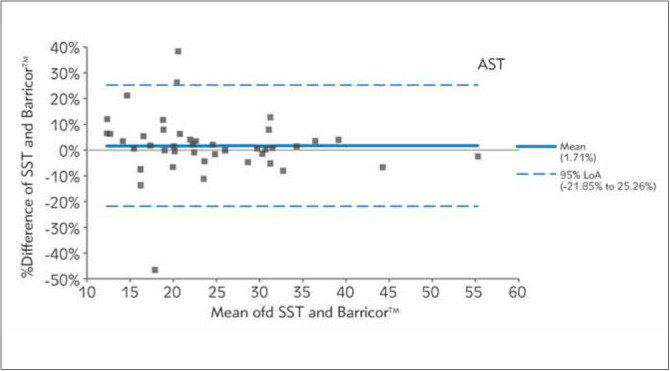
Differences between BarricorTM and Serum separator tube for aspartate aminotransferase values by Bland-Altman analysis (bias% 1.7%). The dashed lines are the limit of agreements (LOA), which correspond to the mean ± 1.96 SD of the difference between the tubes.

**Figure 2:**
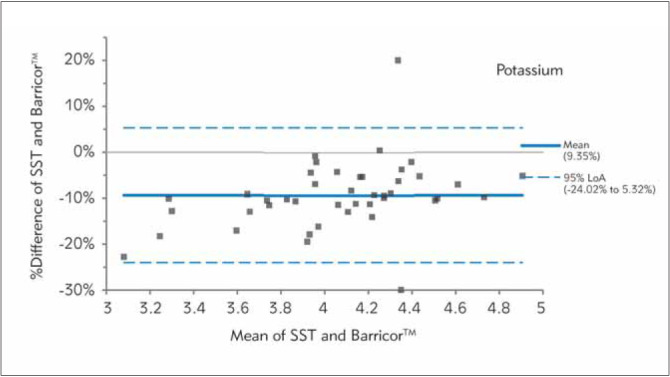
Differences between Barricor^TM^ and Serum separator tube for potassium values by Bland-Altman analysis (bias%=-9.35%). The dashed lines are the limit of agreements (LOA), which correspond to the mean ± 1.96 SD of the difference between the tubes.

**Figure 3:**
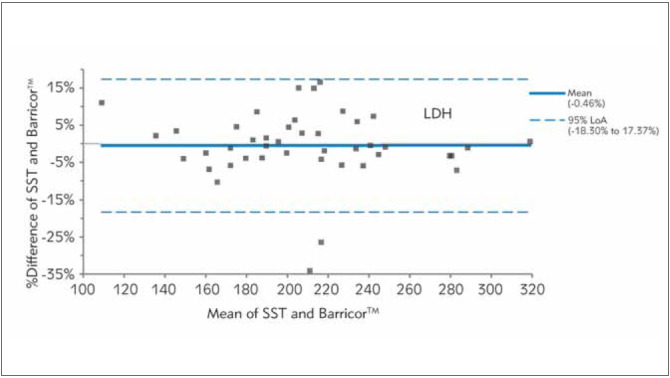
Differences between Barricor^TM^ and Serum separator tube for lactate dehydrogenase (LDH) values by Bland-Altman analysis (bias%=-0.46%). The dashed lines are the limit of agreements (LOA), which correspond to the mean ± 1.96 SD of the difference between the tubes.

**Figure 4:**
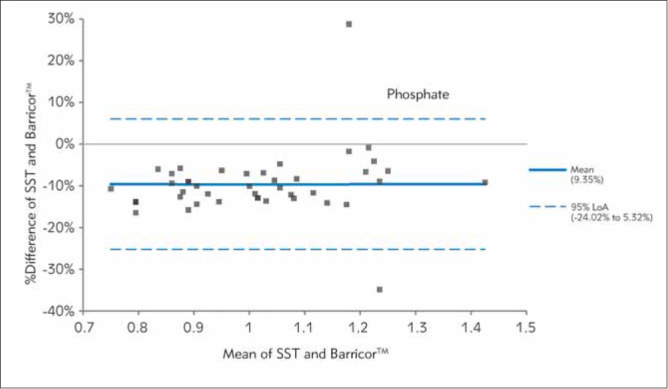
Differences between Barricor^TM^ and Serum separator tube for phosphate values by Bland-Altman analysis (bias%=- 9.59 %). The dashed lines are the limit of agreements (LOA), which correspond to the mean ± 1.96 SD of the difference between the tubes.

**Figure 5:**
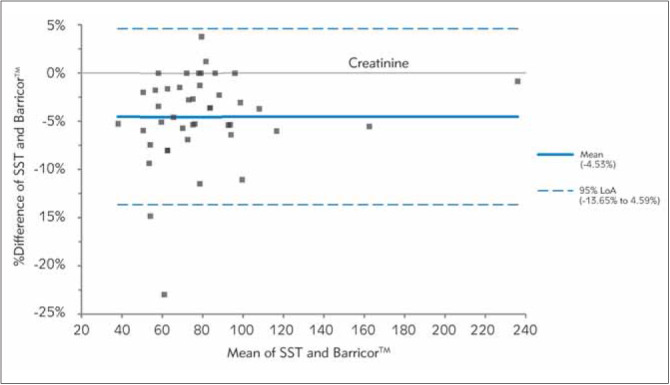
Differences between Barricor^TM^ and Serum separator tube for creatinine values by Bland-Altman analysis (bias%=-4.56 %). The dashed lines are the limit of agreements (LOA), which correspond to the mean ± 1.96 SD of the difference between the tubes.

In Figure 1, Figure 2, Figure 3, Figure 4, Figure 5, Bland-Altman plots demonstrated clinical concordance between the analytes between the tubes, with a mean difference ranging from -9.59 to 1.71%. The mean difference for AST was 1.71% (LOA,-21.85% to 25.26%), with most of the values clustered around the mean. For K, SST measurements were higher, which is reflected by the negative mean difference. For the two analytes (AST and LDH), the mean difference did not exceed the desirable bias. However, the calculated mean difference for K, creatinine, and PO_4_ exceeded the desirable bias.

Table 2 summarises the results for all analytes for SST and Barricor^TM^ and the mean difference (%) at different time intervals. K was only stable up to 48h in both tubes. LDH showed better stability in SST compared to BD Barricor^TM^ (144 h vs 96 h). Phosphate concentrations were more stable in both tubes with the SST having superior stability (144 h vs 120 h). Creatinine had longer stability in both tubes compared to the other analytes tested in this study. Creatinine only exceeded CV_b_, the predefined threshold at 120 h for both tubes. When RCV values were used to determine the acceptable limits, all analytes were acceptable up to 144 h except potassium, which exceeded the RCV at 144 h in SST.

**Table 2:** Mean difference over time for the five analytes SST = serum separator tube, AST = Aspartate aminotransferase, K = Potassium, LDH = Lactate dehydrogenasea Differences are calculated as = (Concentration at point X – Concentration at baseline)/Concentration at baseline *100bTEa = 1.65(0.5xCVI) + 0.25(CVI2 + CVG2)½ using biological variation data from EFLM c RCV = Reference change value.

Mean differences % at difference time points^a^									
Analyte	Tube	24 hrs	48 hrs	72 hrs	96 hrs	120 hrs	144 hrs	TE^a^	RCV^c^ (%)
AST	SST	6.4	7.7	8.2	8.4	8.4	8.8	13.8	±34
	Barricor	4.3	7.5	8.3	10.4	11.6	12.7		
K	SST	0.90	3.15	5.08	7.11	10.66	14.96	4.85	±13.7
	Barricor	1.89	4.77	5.74	6.54	7.63	8.25		
LDH	SST	3	0	-1	-4	-6	-7	7.67	±24
	Barricor	1.1	3.1	4.8	6.8	9.5	11.0		
Phosphate	SST	0.1	0.1	0.9	2.0	4.3	5.1	8.37	±23.5
	Barricor	0.1	1.4	3.9	5.7	6.6	9.4		
Creatinine	SST	2.0	2.0	2.8	2.8	3.4	5.0	7.37	±17.4
	Barricor	0.34	0.81	1.87	2.41	2.77	4.73		

## Discussion

This study evaluated the stability of five analytes in blood samples from the same patient collected almost simultaneously into BD Barricor^TM^ and SST tubes and stored for over seven days, which is the duration of routine sample storage. Barricor^TM^ tubes offer additional advantages to serum tubes. It has shorter centrifugation time and offers complete separation of plasma from cells. Since centrifugation can be done within 9 min vs 30 min in SST, this is likely to improve the turnaround time [5].

For all the analytes tested except potassium, the mean differences between the tubes showed no clinical significance when compared to TEa. Our findings demonstrated lower potassium concentrations in the Barricor^TM^ tube compared to SST. Arslan et al. [5] demonstrated similar findings when the Barricor^TM^ tube was compared to SST. This is due to the release of potassium during the clotting process in the serum separator tube [12]. It is therefore expected that serum potassium values will be significantly higher than plasma. The difference between the tubes is variable and affected by factors such as platelets and red blood cells levels [13], and it may be challenging to derive a correction factor. These two sample tubes may not be used interchangeably in our setting. When using Barricor^TM^ LH plasma to assess potassium concentration, separate reference intervals might be required.

In contrast to the manufacturer claims and other studies findings, the stability of potassium was not superior to SST in our study. Balbás et al. demonstrated superior potassium stability in Barricor tubes when compared to PST II after 12 h [14]. Also, potassium was found to be acceptable up to 72 h in Barricor^TM^ tubes vs 4 h in BD lithium heparin tubes [8]. These studies did not compare Barricor tubes to SST.

Arslan et al. showed a clinically significant bias for AST and LDH when compared to desirable specifications [5]. Our study demonstrated higher mean differences for AST and LDH in the Barricor^TM^ tube compared to SST. Data from other studies suggest that the difference in the Barricor^TM^ bias for AST might be due to the turbulence effect of the mechanical barrier [5] or the higher AST activity may be caused by the presence of heparin, and the magnitude of the elevation is proportional to the concentration of heparin [14] [15]. In contrast, the study by Cadamuro et al. [16]. when compared to other tubes. The difference in LDH is not attributed to different centrifugation conditions [6] [8] [16]. We excluded all the samples that were haemolysed based on a haemolytic index; therefore, the increase in LDH concentrations in our study was not due to artefactual increase. This raised questions about the transferability of reference intervals from other tube types [17] and whether these tubes can be used interchangeably.

In Barricor^TM^ LH plasma, creatinine concentrations were significantly lower than serum. No other studies have demonstrated similar findings. In contrast, other studies that have compared creatinine concentrations between serum and plasma have demonstrated comparable concentrations [5] [18]. However, the stability in both tubes was acceptable up to 144 h. This is vital in our context because our laboratory receives samples from peripheral sites. It is anticipated that by using Barricor tubes, samples can be centrifuged and transported to a central laboratory.

Reference change value offers an objective tool for the evaluation of the significance of the difference between serial results [11]. RCV has been used as a delta check between two measurements from an individual to determine the significant change. To our surprise, when comparing the stability between the tubes using Westgard's desirable specifications for bias and RCV, the results obtained were not similar. In our study, the specifications for TEa were more stringent when compared to RCV. We were able to show that the use of RCV prolonged the stability of the analytes in both tubes. Applying RCV as a criterion in our data might overestimate the stability period. This highlights the need for standardisation of the tools to assess stability and the acceptance criteria to judge any clinically significant deviation.

Participants were recruited in a clinical setting, and thus, the findings are likely to be reflective of everyday practice. This is not the first study internationally, but it carries an element of novelty in that it is the first to be conducted in Africa. Africa and other developing economies face several challenges related to sample processing delays in laboratory medicine.

A limitation of the study is the small sample size, which was limited due to cost implications. However, for method comparison studies, a minimum of 40 samples is required. Another limitation is the lack of inclusion of hospitalised patients. It is not known at this point whether similar results between healthy and diseased individuals can be achieved when the new tube is used. Also, only a few general biochemistry tests were evaluated. These tests were chosen because they are likely to be affected by prolonged contact with cellular material.

## Conclusions

In conclusion, the study demonstrated variability and similarities in analyte concentrations and stability, respectively. For subjects requiring repeat measurement of certain analytes, it is not recommended to use the SST and Barricor^TM^ tubes interchangeably.

Author contributions

All authors were involved in designing the study. SK researched the literature, analysed the data, and wrote the first draft of the manuscript. SF analysed the samples. All authors reviewed and edited the manuscript and approved the final version of the manuscript.

*Funding*. Becton Dickinson provided the collection tubes. The funding organisation played no role in the design of the study, choice of enrolled patients, review, and interpretation of data, preparation of the manuscript.

*Acknowledgements. *The authors would like to acknowledge the patients at Lenasia District Hospital who agreed to participate in this study, the laboratory staff for analysing the samples, and the District Research Committee for allowing us to conduct the study at the facility.

## Conflict of interest statement

All the authors declare that they have no conflict of interest in this work.
